# Biobased Engineering Thermoplastics: Poly(butylene 2,5-furandicarboxylate) Blends

**DOI:** 10.3390/polym11060937

**Published:** 2019-05-29

**Authors:** Niki Poulopoulou, George Kantoutsis, Dimitrios N. Bikiaris, Dimitris S. Achilias, Maria Kapnisti, George Z. Papageorgiou

**Affiliations:** 1 Chemistry Department, University of Ioannina, P.O. Box 1186, 45110 Ioannina, Greece; nikki_p@windowslive.com (N.P.); gkantoutsis@hotmail.com (G.K.); 2 Laboratory of Polymer and Dyes Chemistry and Technology, Department of Chemistry, Aristotle University of Thessaloniki, GR-541 24 Thessaloniki, Macedonia, Greece; axilias@chem.auth.gr; 3 Department of Food Science and Technology, International Hellenic University, PO Box 141, GR-57400 Thessaloniki, Greece; marikapn@yahoo.gr

**Keywords:** biobased polymers, renewable resources, FDCA, polymer blends, poly(butylene 2,5-furandicarboxylate), polyesters

## Abstract

Poly(butylene 2,5-furandicarboxylate) (PBF) constitutes a new engineering polyester produced from renewable resources, as it is synthesized from 2,5-furandicarboxylic acid (2,5-FDCA) and 1,4-butanediol (1,4-BD), both formed from sugars coming from biomass. In this research, initially high-molecular-weight PBF was synthesized by applying the melt polycondensation method and using the dimethylester of FDCA as the monomer. Furthermore, five different series of PBF blends were prepared, namely poly(l-lactic acid)–poly(butylene 2,5-furandicarboxylate) (PLA–PBF), poly(ethylene terephthalate)–poly(butylene 2,5-furandicarboxylate) (PET–PBF), poly(propylene terephthalate)–poly(butylene 2,5-furandicarboxylate) (PPT–PBF), poly(butylene 2,6-naphthalenedicarboxylate)-poly(butylene 2,5-furandicarboxylate) (PBN–PBF), and polycarbonate–poly(butylene 2,5-furandicarboxylate) (PC–PBF), by dissolving the polyesters in a trifluoroacetic acid/chloroform mixture (1/4 *v*/*v*) followed by coprecipitation as a result of adding the solutions into excess of cold methanol. The wide-angle X-ray diffraction (WAXD) patterns of the as-prepared blends showed that mixtures of crystals of the blend components were formed, except for PC which did not crystallize. In general, a lower degree of crystallinity was observed at intermediate compositions. The differential scanning calorimetry (DSC) heating scans for the melt-quenched samples proved homogeneity in the case of PET–PBF blends. In the remaining cases, the blend components showed distinct T_g_s. In PPT–PBF blends, there was a shift of the T_g_s to intermediate values, showing some partial miscibility. Reactive blending proved to improve compatibility of the PBN–PBF blends.

## 1. Introduction

Biobased polymers are those that can be derived directly from biomass or can be synthesized from monomers derived from biomass. Such polymers are gaining increasing interest from both academic and industrial points of view [1,2]. The production capacity of biobased polymers is expected to reach around 12 million tons by 2020 [3]. As an example, 2,5-furandicarboxylic acid (2,5-FDCA) is considered a potential biobased replacement for terephthalic acid, which is the basis for the production of polyesters such as poly(ethylene terephthalate) (PET) and poly(propylene terephthalate) (PPT) [4,5,6,7]. Three compounds—2,5-FDCA, 5-hydroxymethyl-furfural (5-HMF), and 2,5-dimethylfuran (2,5-DMF)—are considered the “sleeping giants” of renewable intermediate chemicals [8,9].

Much effort is put into selective aerobic oxidation and catalytic conversion of HMF or furfural to obtain FDCA [8]. Furan-based polyesters were first reported by Gandini and Kelly and Moore [10,11]. However, today, it is well known that almost all furanoate polyesters are prepared using 2,5-FDCA and different diols, and their mechanical, gas barrier, and thermal properties were reported in the literature [4,9]. For the synthesis of these polyesters, as well as their copolymers, in addition to the typical melt polycondensation methods, ring-opening polymerization and lipase-catalyzed (enzymatic) methods were also proposed [12,13,14,15].

Poly(butylene 2,5-furandicarboxylate) (PBF), the biobased poly(butylene terephthalate) (PBT) counterpart, is produced by polymerization of 1,4-butanediol (BD) with FDCA [16,17]. Zhu et al. studied the crystal structure and mechanical properties of PBF [18] and found that it shows similar mechanical properties and crystal structure to PBT. The glass transition temperature of PBF was found to be 36 °C, and the melting point is about 170 °C; moreover, two crystal modifications, α and β, were reported for PBF [19]. The more stable α-crystal modification is characterized by a triclinic unit cell with a = 4.78 Å, b = 6.03 Å, c = 12.3 Å, α = 110.1°, β = 121.1°, γ = 100.6° [20]. The crystallization process and melting behavior of PBF was studied by Papageorgiou et al. [21]. Aliphatic–aromatic copolyesters related to PBF, such as poly(butylene adipate-*co*-butylene furandicarboxylate) or poly(butylene succinate-*co*-butylene furandicarboxylate), were studied in an attempt to explore probabilities of producing biodegradable furan-based polyesters [22,23,24]. A few more cases of PBF-related copolymers, such as poly(butylene 2,5-furanoate/diglycolate), blocky poly(ε-caprolactone-*co*-butylene 2,5-furandicarboxylate), copolymers based on PBF and poly(ethylene glycol), poly(butylene 2,5-furandicarboxylate-*co*-terephthalate), and poly(carbonate-*co*-ester), were reported in the literature [25,26,27,28,29,30,31].

In recent decades, much attention was paid to biobased polyesters, including aliphatic biodegradable poly(lactic acid) (PLA) or poly(butylene succinate) (PBS) [32,33,34]. PBF as an aromatic polyester has advantageous thermal, mechanical, and gas barrier properties compared with biodegradable polyesters. In fact, there is a real need for aromatic biobased polymers, as their properties can better fulfill the demands for applications. On the other hand, aromatic polymers are by virtue non-biodegradable.

Despite the favorable properties of furanoates, PBF has a low T_g_ compared to other aromatic polymers and it is also non-biodegradable. Furthermore, it shows a rather slow crystallization rate and moderate melting temperature. Additionally, industrialization of furanoate polyesters is not the easiest task, and total replacement of terephthalate polyesters by furanoates, if possible, is not expected to occur soon [3]. Thus, it would be of interest to find ways to overcome these drawbacks which limit the industrial applications of PBF.

In general, polymers can be modified by copolymerization, blending, or incorporation of fillers. In contrast to PBF-related copolymers, on which a series of works were already conducted, blends of PBF are yet to be fully characterized, and only a few papers on this topic appeared in open literature [35,36,37,38].

In this work, a series of blends based on PBF combinations with significant polyesters such as poly(lactic acid) (PLA), poly(ethylene terephthalate) (PET), poly(propylene terephthalate) (PPT), poly(butylene naphthalate) (PBN), and polycarbonate (PC) were prepared and characterized with respect to homogeneity and miscibility. Although several studies were conducted on polymer blends based on PLA or PET and the other polymers, this is the very first work dedicated to blends with PBF. The objective of this work was to explore blending of PBF with the above polymers aiming at improving biodegradability (PLA–PBF blends), increasing T_g_ (PC–PBF), as well as crystallization, and facilitating the industrialization of PBF (PET–PBF and PPT–PBF blends).

## 2. Materials and Methods

### 2.1. Synthesis of Polyesters

The main compound, 2,5-furan dicarboxylic acid (97% purity), was purchased from Sigma-Aldrich Chemical Co (Chemie GmbH, Steinheim, Germany). Tetrabutyltitanate (TBT) catalyst of analytical grade and 1,4-butanediol of analytical grade, used in polyester synthesis, were purchased from Sigma-Aldrich Chemical Co (Chemie GmbH, Steinheim, Germany). All other materials and solvents used were of analytical grade. Solid-state polycondensation (SSP) was subsequently applied to produce polymers of high molecular weight.

High-molecular-weight poly(butylene 2,5-furandicarboxylate) (PBF) was synthesized by applying melt polycondensation following the general procedure described in our previous study [21]. PET, PPT, and PBN were also prepared, as described in our previous studies, via the melt polycondensation procedure [39,40]. PLA, with an average molecular weight of Mw = 20,000 Da and a polydispersity index of about 1.3, and poly(bisphenol A carbonate), with an average Mw of about 45,000 Da, were purchased from Sigma-Aldrich Chemical Co.

### 2.2. Preparation of Polymer Blends

Polymer blends of the thermoplastic polyesters were prepared by dissolving the corresponding polymer pairs in a mixture of trifluoroacetic acid and chloroform (1/4 *v*/*v*). The solutions were poured into an excess of methanol, and the blends were obtained as the precipitate. Several blends with varying compositions were prepared. Solution mixing was selected for the preparation of blends in order to avoid any possible transesterification reactions occurring at elevated temperatures during melt-mixing.

### 2.3. Characterization Methods

#### 2.3.1. Intrinsic Viscosity Measurements

Intrinsic viscosity (IV) [η] measurements were performed using an Ubbelohde viscometer (Schott Gerate GMBH, Hofheim, Germany) at 30 °C in a mixture of phenol/1,1,2,2-tetrachloroethane (60/40, *w*/*w*). IV values for neat polymers were found to be 0.65, 0.59, 0.62, and 0.67 dL/g for PBF, PET, PPT, and PBN, respectively. Additionally, the intrinsic viscosity measurements for the blends PLA–PBF 50/50, PET–PBF 50/50, PPT–PBF 50/50, PBN–PBF, and PC–PBF 50/50 were estimated at 0.68, 0.60, 0.59, 0.63, and 0.69 dL/g, respectively.

#### 2.3.2. Differential Scanning Calorimetry (DSC)

The thermal behavior of the blends was studied using a Perkin Elmer Diamond DSC (PerkinElmer Corporation, Waltham MA, USA) upgraded to DSC 8500, combined with an Intracooler IIP cooling system. Samples of about 5 mg were used. The blends were firstly heated at 20 °C/min up to 30 °C above the higher melting temperature and then quenched to −30 °C, before reheating at a rate of 20 °C/min, in order to observe the glass transition, cold crystallization, and melting of the amorphous samples. For polyesters, reactive blending is an industrial process that involves melt-mixing in an extruder/internal mixer, at temperatures higher than the melting temperatures of all constituents [41]. To simulate reactive blending, the blends were initially prepared from solution, as described above, and were subsequently melt-mixed within the DSC pans. More specifically, for reactive blending experiments, the blends were scanned at a rate of 20 °C/min up to a predetermined temperature (well above the melting points of both components), where they were held for a specific time in each test, before quenching to −30 °C. The quenched samples were subsequently heated at 20 °C/min, starting from a temperature at least 30 °C below the lower T_g_ of the polymers. For the evaluation of the glass transition, tangents were drawn carefully on the heat flow curve at temperatures above and below the glass transition, and the T_g_ was obtained as the point of intersection of the bisector of the angle between the tangents with the heat flow curve. The intersection of these tangents with that of the part corresponding to the transition were used as *T_g,onset_* and *T_g,end_*.

#### 2.3.3. X-ray Diffraction

Powder X-ray diffraction measurements of samples were performed using Rigaku Mini Flex 600 (Rigaku Co., Tokyo, Japan) with Bragg–Brentano geometry (θ–2θ), using CuKa radiation (k = 0.154 nm) in the angle 2θ range from 5° to 60°. The slit was 1.25°, the accuracy was ± 0.05° and the scanning speed was 1 min^−1^.

#### 2.3.4. Polarizing Light Microscopy (PLM)

A polarizing light microscope (Nikon, Optiphot-2, Melville, NY, USA), equipped with a Linkam THMS600 heating stage, a Linkam TP 91 (Linkam Scientific Instruments Ltd., Surrey, UK) control unit, and a JenopticProgRes C10Plus camera (Jenoptik Optical Systems GmbH, Jena, Germany), was used for PLM observations.

## 3. Results and Discussion

### 3.1. Synthesis of PBF

**Figure 1:**
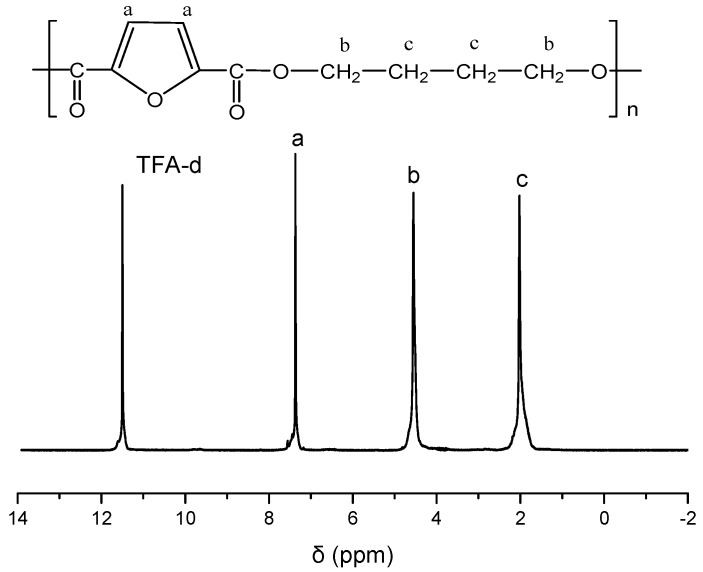
^1^H NMR spectrum of poly(butylene 2,5-furandicarboxylate) (PBF).

PBF was synthesized by applying the melt polycondensation method, and it has an intrinsic viscosity value 0.65 dL/g. The successful synthesis of PBF was verified by the collected ^1^H NMR spectrum of the sample, as shown in Figure 1. The other polyesters exhibited similar intrinsic viscosity values, equal to 0.59, 0.62, and 0.67 dL/g for PET, PPT, and PBN, respectively.

### 3.2. PLA–PBF Blends

**Figure 2:**
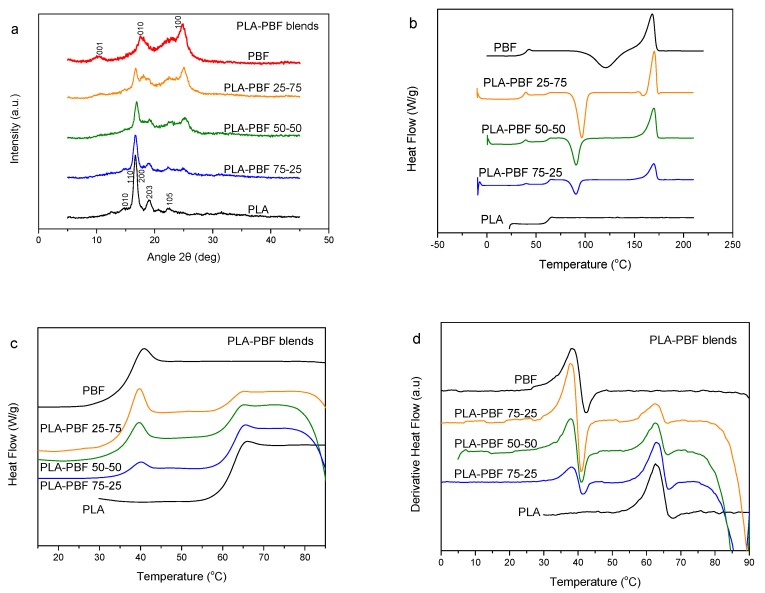
(**a**) Wide-angle X-ray diffraction (WAXD) patterns, (**b**) differential scanning calorimetry (DSC) traces of the melt-quenched samples upon heating, (**c**) details in the T_g_ region, and (**d**) derivative heat flow thermograms for the poly(lactic acid) (PLA)–PBF blends.

PLA is a biodegradable polyester of special interest [42]. It would be interesting to exploit its advantages in blends with the PBF resin, due to their potential uses in biobased/biodegradable food packaging. PLA–PBF blends were prepared, and the wide-angle X-ray diffraction (WAXD) patterns of the as-prepared samples by precipitation are shown in Figure 2a. Both PLA and PBF crystallized. The DSC traces of the blends after melt-quenching are presented in Figure 2b. As can be seen, dual glass transitions appear in the DSC scans, showing that the two polymers are essentially not miscible. To better study this behavior, the magnified traces of Figure 2c and the thermograms of the derivative of heat flow vs. temperature of Figure 2d can be used. As a matter of fact, in these figures there does not appear any variation of the glass transition temperatures of the blend components. This is proof that miscibility is absent. In the traces of Figure 2b, it is also clear that the cold crystallization behavior is different to that of neat PBF, as, in the blends, the cold-crystallization peak is positioned at a much lower temperature compared to that for the neat PBF. In contrast, neat PLA did not show any cold crystallization upon heating. The cold crystallization enthalpy for blends decreased with increasing PLA content, indicating that PLA does not crystallize significantly, although its crystallization cannot be totally precluded.

### 3.3. PET–PBF Blends

**Figure 3:**
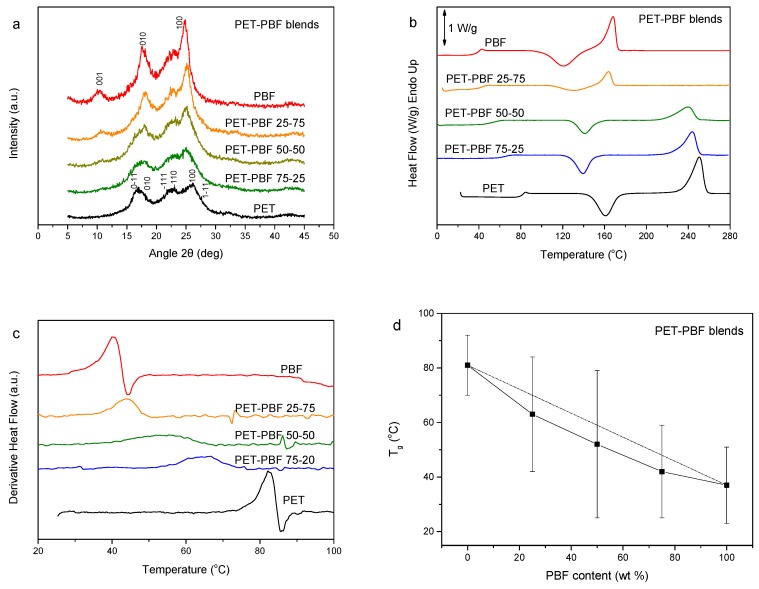
(**a**) WAXD patterns, (**b**) DSC traces of the melt-quenched samples upon heating, (**c**) derivative heat flow thermograms, and (**d**) variation of T_g_ and glass transition width with PBF content, for the poly(ethylene terephthalate) (PET)–PBF blends.

PET is a giant in the thermoplastic polyester industry due to its favorable properties [43]. Its blends with PBF constitutes a challenge. The WAXD patterns of the PET–PBF blends can be viewed in Figure 3a. It was revealed that mixtures of the PET and PBF crystals are formed by precipitation of the blends from solution. In other words, the polyesters crystallize separately. DSC traces of Figure 3b show a single composition-dependent glass transition, which manifests a dynamic homogeneity of the blends. This is better exhibited in the inset of Figure 3b, where the enlarged DSC traces are shown. In the heat flow derivative curves, one can clearly see the single composition-dependent glass transition as a single peak (Figure 3c). However, the peak width is large for blends with intermediate compositions. Figure 3c shows the variation of T_g_ and the glass transition temperature width with blend composition. This fact might be evidence that the components are not completely miscible. The increased transition width in intermediate compositions is clear. However, as shown by Lodge, even miscible blends show dual T_g_s. The fact that the T_g_ values are lower than those for the diagonal is further proof of the absence of complete miscibility. Thus, the PET–PBF blends are partially miscible. In the DSC traces of Figure 3b, it is also clear that, for a PET content of more than 25 wt.%, only PET crystallized, as there was no melting peak for PBF. In contrast, in the case of the PET–PBF 25/75, only PBF crystallized, as proven by the appearance of the PBF melting peak and absence of the PET one.

### 3.4. PPT–PBF Blends

**Figure 4:**
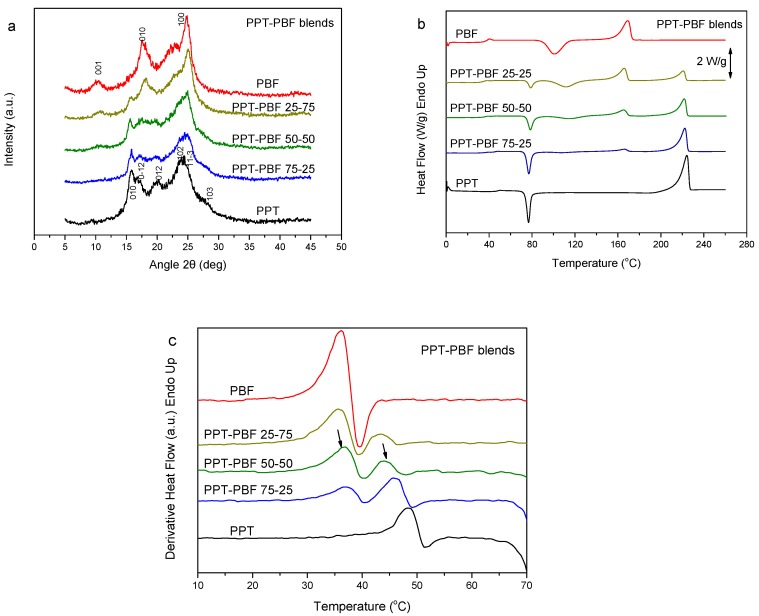
(**a**) WAXD patterns, (**b**) DSC traces of the melt-quenched samples upon heating, and (**c**) derivative heat flow thermograms of the poly(propylene terephthalate) (PPT)–PBF blends.

In this section, the PPT–PBF blends are discussed. As PPT is considered a biobased polyester of 1,3-propanediol (PDO) [44,45], blending with the biobased PBF results in novel fully biobased materials. The WAXD patterns of the blends revealed the crystallizability of both components in the blends during precipitation from solution (Figure 4a). It seems that mixtures of the crystals of the two polymers are formed, and not crystals of the blend. The DSC thermograms for the melt-quenched PPT–PBF blend samples showed dual glass transitions (Figure 4b). An exception was found in case of the PPT–PBF 80/20, for which a single glass transition was observed (not shown here). This is also observed in the derivative heat flow thermograms (Figure 4c). A variation of the T_g_ values was also evident with blend composition. Thus, the PPT–PBF blends exhibit some partial miscibility.

### 3.5. PBN–PBF Blends

**Figure 5:**
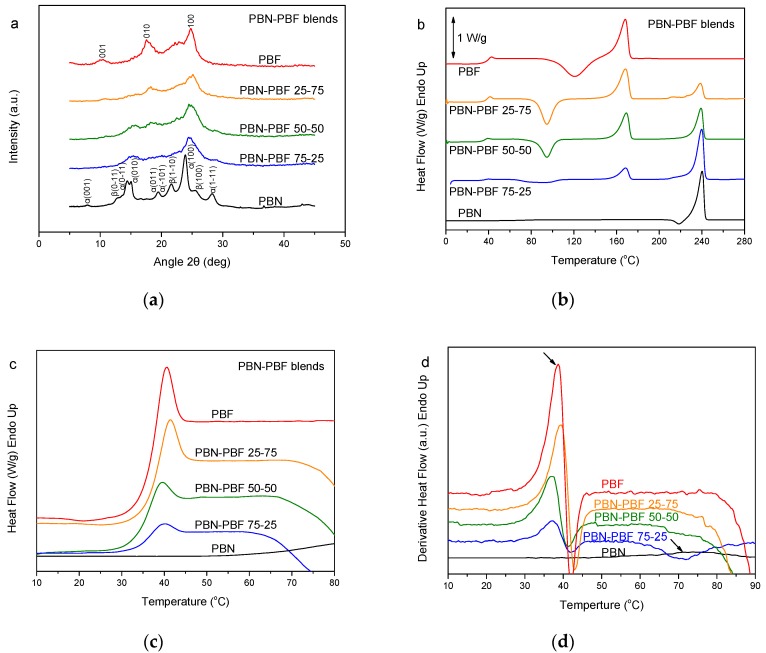
(**a**) WAXD patterns, (**b**) DSC traces of the melt-quenched samples upon heating, (**c**) zoom of the DSC traces in the region of T_g_, and (**d**) derivative heat flow for the poly(butylene naphthalate) (PBN)–PBF blends.

The last case of blends studied in this work was that of PBN–PBF ones. PBN is a very fast-crystallizing engineering polyester [46]. Its crystallization rates are much faster than those for PBF [47]. The WAXD patterns showed that PBN–PBF blends form mixtures of crystals of PBN and PBF. However, it is true that, in the WAXD patterns of the blends, the peaks were significantly broader with lower intensity (Figure 5a). This is proof of reduced crystal size and degree of crystallinity for the blends. The DSC traces for these samples revealed a significant trend of crystallization for both the polymers (Figure 5b). In fact, PBN could not be obtained in the glassy amorphous state, despite melt-quenching. Finally, as can be seen in Figure 5c,d, showing details in the glass transition region and the derivative heat flow, respectively, the glass transition for the PBF is at the same temperature. After all, the PBN–PBF blends are immiscible.

**Figure 6:**
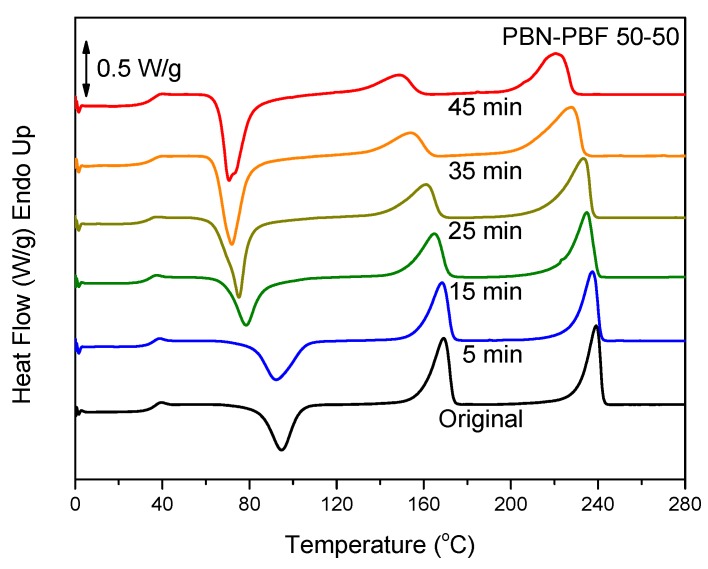
DSC traces of the melt-quenched PBN–PBF 50/50 samples after reactive blending for the indicated times.

To improve homogeneity and miscibility of polyester blends, reactive blending can be applied [37]. The process involves melt-mixing at high temperatures for relatively prolonged durations, so that transesterification reactions can take place. The latter lead to block copolymers and compatibilization. In case of longer times of melt-mixing, especially at elevated temperatures well above the melting temperatures of the blend components, random copolymers can be obtained. This was proven in our previous study [37]. Detailed studies of reactive blending of the particular blends of PBF will follow. In this work, the preliminary study of the reactive blending in the case of PBN–PBF blends with DSC is presented. In fact, the reactive blending was simulated using small blend amounts in the DSC sample pan and heating the samples to 300 °C. Figure 6 shows the effect of time of melt-mixing at 300 °C on the thermal behavior of the corresponding PBN–PBF 50/50 blend samples after melt-quenching. As can be seen, only the glass transition for PBN is apparent. This is because of the fast crystallization of PBN, but also due to the fact that cold crystallization occurred at temperatures in the vicinity of the glass transition for PBN. However, it should be noted that the cold crystallization peak decreased in peak temperature and, at the same time, it increased in enthalpy of crystallization. The increase in the ΔC_p_ of PBF is also obvious, and this also shows that the samples always showed a lower degree of crystallinity with increasing melt-mixing time. After 45 min of reactive blending, although dual melting peaks, one for the PBF and a second for the PBN component, are still present in the DSC heating scans for the melt-quenched sample, the degree of crystallinity is close to 0, as can be realized by comparing the enthalpies of fusion of PBF and PBN and the enthalpy of cold crystallization. This means that the copolymerization took place to some extent during reactive blending, and this drastically limited the crystallization rates of PBN or PBN-rich copolymer chains. Furthermore, the melting point depression is clear with increasing reactive blending time, also verifying the hypothesis of copolymerization. As a matter of fact, the times to achieve this were very long. However, from the practical point of view, it is important that some compatibilization can be achieved even after 15 min of reactive blending or after even shorter times of reactive blending.

### 3.6. PC–PBF Blends

**Figure 7:**
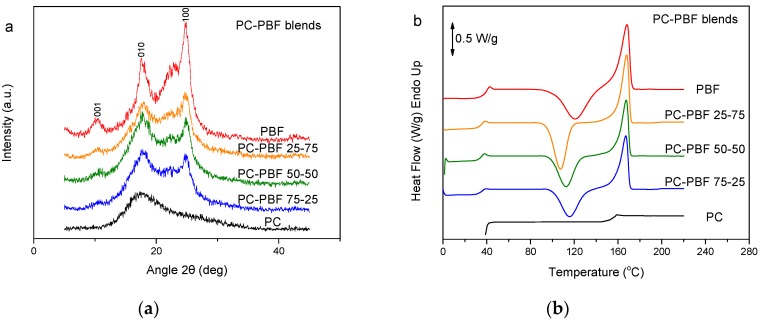

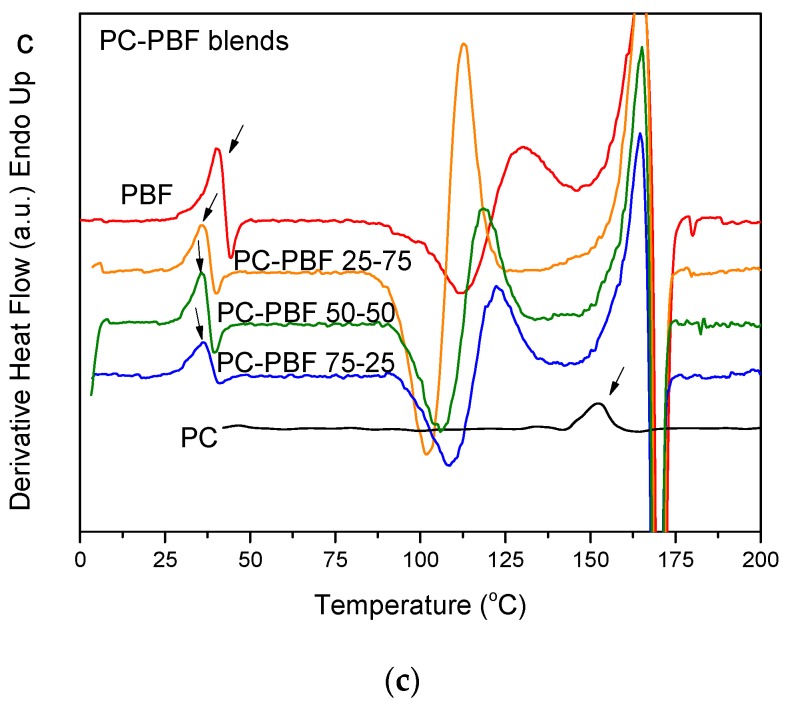
(**a**) WAXD patterns, (**b**) DSC traces of the melt-quenched samples upon heating, and (**c**) derivative heat flow for the polycarbonate (PC)–PBF blends.

In contrast to fast-crystallizing PBN, PC is essentially amorphous [48]. Figure 7a shows the WAXD patterns of PC, PBF, and their blends. The patterns for the blends revealed that the PBF component crystallized. However, PBF showed lower crystallinity in the blends. Also, there was differentiation in the peak heights, showing different crystallites orientation. Figure 7b shows the DSC thermograms of the melt-quenched PC–PBF blends in comparison to neat PC and neat PBF. No shift to higher temperatures can be seen in the T_g_ of PBF. In contrast, the T_g_ for PBF is a little lower in the blends, most probably because some small amount of solvent remained in the blends (Figure 7c). The glass transition of PC is overlapped by the melting peak of PBF. The findings are consistent with immiscible blends. In every blend composition, PBF suffered cold crystallization. In fact, the cold crystallization for the blends appeared at a lower temperature compared to that for neat PBF. However, this could be due to some residual solvent effects and not because of the effects of mixing.

### 3.7. Calculation of the Solubility Parameters of the Polymers

To further evaluate the miscibility of the polymer pairs, the solubility parameter δ values were calculated. The cohesive energy may be divided into three parts, corresponding to the three types of interaction forces.

(1) $$E_{coh}=E_{d}+E_{p}+E_{h},$$

where *E_d_* is the contribution of dispersion forces, *E_p_* is the contribution of polar forces, and *E_h_* is the contribution of hydrogen bonding.

The corresponding equation for the solubility parameter is

(2) $$\delta^{2}=\delta_{d}^{2}+\delta_{p}^{2}+\delta_{h}^{2}.$$

Firstly, the values for molar volume were calculated. Then, the solubility parameters were calculated using the component group contributions (method Hoftyzer–van Krevelen) [49].

The results can be found in Table 1. The values for PBF and PET are quite close to each other. Thus, they are expected to show some miscibility.

### 3.8. Polarized Light Microscopy Study

**Figure 8:**
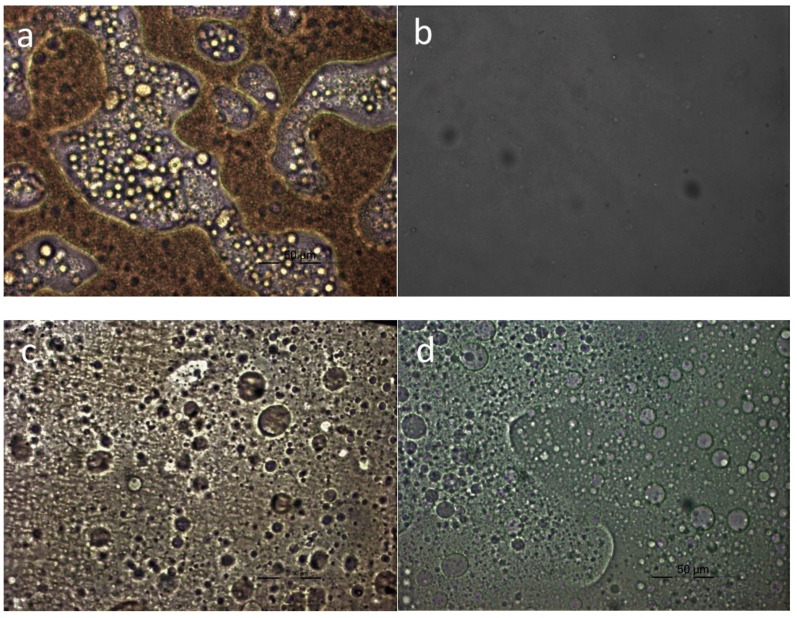
Polarized light microscopy (PLM) photographs for PBF blends in the melt phase: (**a**) PLA–PBF 50/50; (**b**) PET–PBF 50/50; (**c**) PPT–PBF 50/50; (**d**) PC–PBF 50/50.

PLM was finally used to directly observe the appearance of the blends in the melt state and the morphology generated after crystallization of the components. As can be seen in Figure 8, the blends showed phase separation in the melt phase except in the case of PET–PBF mixtures. In the case of the melt of the PET–PBF blend, shown in Figure 8b, a rather homogeneous system was verified. Therefore, these observations are in line with all the findings reported in the previous sections.

## 4. Conclusions

PBF was synthesized by applying melt polycondensation and used to prepare blends with several polyesters. PLA–PBF, PBN–PBF, and PC–PBF blends were found to be immiscible. PET–PBF blends showed a single composition-dependent T_g_ in every blend composition in the DSC heating thermograms for melt-quenched samples, indicating dynamic homogeneity and probably miscibility of the two blend components. For the PPT–PBF blends, dual T_g_s were observed, but a shift of both T_g_s was also verified at intermediate temperatures. This is an indication of partial miscibility. Particularly, in the case of the PPT–PBF 80/20, a single T_g_ was found, proving dynamic homogeneity of the particular mixture. Moreover, for the immiscible blends, reactive blending, as exemplified in the case of PBN–PBF blends, improved the homogeneity of the mixtures.

## Figures and Tables

**Table 1:** Molar volumes and solubility parameter values for the polymers used in blends. PBF—poly(butylene 2,5-furandicarboxylate); PLA—poly(lactic acid); PET—poly(ethylene terephthalate); PPT—poly(propylene terephthalate); PBN—poly(butylene naphthalate); PC—polycarbonate.

Polymer	V (cm^3^/mol)	δ^2^ (MJ/m^3^)^1/2^
PBF	158.4	22.2
PLA	60.7	19.9
PET	144.2	22.0
PPT	159.2	21.5
PBN	223.5	20.2
PC	212.0	20.9

## References

[1] Zhu Y., Romain C., Williams C.K. (2016). Sustainable polymers from renewable resources. Nature.

[2] Vilela C., Sousa A.F., Fonseca A.C., Serra A.C., Coelho J.F.J., Freire C.S.R., Silvestre A.J.D. (2014). The quest for sustainable polyesters—Insights into the future. Polym. Chem..

[3] Papageorgiou G.Z., Papageorgiou D.G., Terzopoulou Z., Bikiaris D.N. (2016). Production of bio-based 2,5-furan dicarboxylate polyesters: Recent progress and critical aspects in their synthesis and thermal properties. Eur. Polym. J..

[4] Dijkman W.P., Groothuis D.E., Fraaije M.W. (2014). Enzyme-Catalyzed oxidation of 5-Hydroxymethylfurfural to Furan-2,5-dicarboxylic acid. Angew.Chem..

[5] Dick G.R., Frankhouser A.D., Banerjee A., Kanan M.W. (2017). A scalable carboxylation route to furan-2,5-dicarboxylic acid. Green Chem..

[6] Teles J.H. (2019). Across the Board: J. Henrique Teles. ChemSusChem.

[7] Hayashi E., Yamaguchi Y., Kamata K., Tsunoda N., Kumagai Y., Oba F., Hara M. (2019). Effect of MnO_2_ Crystal Structure on Aerobic Oxidation of 5-Hydroxymethylfurfural to 2,5-Furandicarboxylic Acid. J. Am. Chem. Soc..

[8] Tong X., Ma Y., Li Y. (2010). Biomass into chemicals: Conversion of sugars to furan derivatives by catalytic processes. Appl. Catal. A Gen..

[9] Sousa A.F., Vilela C., Fonseca A.C., Gruter G.-J.M., Coelho J.F.J., Silvestre A.J.D., Matos M., Freire C.S.R. (2015). Biobased polyesters and other polymers from 2,5-furandicarboxylic acid: A tribute to furan excellency. Polym. Chem..

[10] Gandini A. (1977). The behavior of furan derivatives in polymerization reactions. Adv. Polym. Sci..

[11] Moore J.A., Kelly J.E. (1978). Polyesters Derived from Furan and Tetrahydrofuran Nuclei. Macromolecules.

[12] Morales-Huerta J.C., Martínez de Ilarduya A., Muñoz-Guerra S. (2016). Poly(alkylene 2,5-furandicarboxylate)s (PEF and PBF) by ring opening polymerization. Polymer.

[13] Jiang Y., Woortman A.J.J., Alberda van Ekenstein G.O.R., Loos K. (2015). A biocatalytic approach towards sustainable furanic–aliphatic polyesters. Polym. Chem..

[14] Morales-Huerta J.C., Ciulik C.B., Martínez de Ilarduya A., Muñoz-Guerra S. (2017). Fully bio-based aromatic–aliphatic copolyesters: Poly(butylene furandicarboxylate-co-succinate)s obtained by ring opening polymerization. Polym. Chem..

[15] Jiang Y., Woortman A.J.J., Alberda van Ekenstein G.O.R., Petrovic D.M., Loos K. (2014). Enzymatic Synthesis of Biobased polyesters using 2,5-Bis(hydroxymethyl)furan as the building block. Biomacromolecules.

[16] Ma J., Pang Y., Wang M., Xu J., Ma H., Nie X. (2012). The copolymerization reactivity of diols with 2,5-furandicarboxylic acid for furan-based copolyester materials. J. Mater. Chem..

[17] Knoop R.J., Vogelzang W., Haveren J., Es D.S. (2013). High molecular weight poly(ethylene-2,5-furanoate); critical aspects in synthesis and mechanical property determination. J. Polym. Sci. Part A Polym. Chem..

[18] Zhu J., Cai J., Xie W., Chen P.-H., Gazzano M., Scandola M., Gross R.A. (2013). Poly(butylene 2,5-furan dicarboxylate), a Biobased Alternative to PBT: Synthesis, Physical Properties, and Crystal Structure. Macromolecules.

[19] Ma J., Yu X., Xu J., Pang Y. (2012). Synthesis and crystallinity of poly(butylene 2,5-furandicarboxylate). Polymer.

[20] Soccio M., Martínez-Tong D.E., Alegría A., Lotti N. (2017). Molecular dynamics of fully biobased poly(butylene 2,5-furanoate) as revealed by broadband dielectric spectroscopy. Polymer.

[21] Papageorgiou G.Z., Tsanaktsis V., Papageorgiou D.G., Exarhopoulos S., Papageorgiou M., Bikiaris D.N. (2014). Evaluation of polyesters from renewable resources as alternatives to the current fossil-based polymers. Phase transitions of poly (butylene 2,5-furan-dicarboxylate). Polymer.

[22] Wu L., Mincheva R., Xu Y., Raquez J.M., Dubois P. (2013). High Molecular Weight Poly(butylene succinate-co-butylene furandicarboxylate) Copolyesters: From Catalyzed Polycondensation Reaction to Thermomechanical Properties. Biomacromolecules.

[23] Wu B., Xu Y., Bu Z., Wu L., Li B.-G., Dubois P. (2014). Biobasedpoly(butylene 2,5-furandicarboxylate) and poly(butylene adipate-co-butylene 2,5-furandicarboxylate)s: From synthesis using highly purified 2,5-furandicarboxylic acid to thermo-mechanical properties. Polymer.

[24] Zhou W., Wang X., Yang B., Xu Y., Zhang W., Zhang Y., Ji J. (2013). Synthesis, physical properties and enzymatic degradation of bio-based poly(butylene adipate-co-butylene furandicarboxylate) copolyesters. Polym. Degrad. Stab..

[25] Jacquel N., Saint-Loup R., Pascault J.-P., Rousseau A., Fenouillot F. (2015). Bio-based alternatives in the synthesis of aliphatic-aromatic polyesters dedicated to biodegradable film applications. Polymer.

[26] Soccio M., Costa M., Lotti N., Gazzano M., Siracusa V., Salatelli E., Manaresi P., Munari A. (2016). Novel fully biobased poly(butylene 2,5-furanoate/diglycolate) copolymers containing ether linkages: Structure-property relationships. Eur. Polym. J..

[27] Morales-Huerta J.C., Martınez de Ilarduya A., Munoz-Guerra S. (2018). Blocky poly(ε-caprolactone-co-butylene 2,5-furandicarboxylate) copolyesters via enzymatic ring opening polymerization. J. Polym. Sci. A Polym. Chem..

[28] Morales-Huerta J.C., Martínez de Ilarduya A., Muñoz-Guerra S. (2016). Sustainable Aromatic Copolyesters via Ring Opening Polymerization: Poly(butylene 2,5-furandicarboxylate-co-terephthalate)s. ACS Sustain. Chem. Eng..

[29] Sousa A.F., Guigo N., Pożycka M., Delgado M., Soares J., Mendonca P.V., Coelho J.F.J., Sbirrazzuoli N., Silvestre A.J.D. (2018). Tailor design of renewable copolymers based on poly(1,4-butylene 2,5-furandicarboxylate) and poly(ethylene glycol) with refined thermal properties. Polym. Chem..

[30] Zhou W., Zhang Y., Xu Y., Wang P., Gao L., Zhang W., Ji J. (2014). Synthesis and characterization of bio-based poly(butylene furandicarboxylate)-b-poly(tetramethylene glycol) copolymers. Polym. Degrad. Stab..

[31] Cai X., Yang X., Zhang H., Wang G. (2018). Aliphatic-aromatic poly(carbonate-co-ester)s containing biobased furan monomer: Synthesis and thermo-mechanical properties. Polymer.

[32] Kuang T., Chen F., Chang L., Zhao Y., Fu D., Gong X., Peng X. (2017). Facile preparation of open-cellular porous poly (l-lactic acid) scaffold by supercritical carbon dioxide foaming for potential tissue engineering applications. Chem. Eng. J..

[33] Kuang T., Ju J., Yang Z., Geng L., Peng X. (2018). A facile approach towards fabrication of lightweight biodegradable poly (butylene succinate)/carbon fiber composite foams with high electrical conductivity and strength. Compos. Sci. Technol..

[34] Kuang T., Li K., Chen B., Peng X. (2017). Poly(propylene carbonate)-based in situ nanofibrillar biocomposites with enhanced miscibility, dynamic mechanical properties, rheological behavior and extrusion foaming ability. Compos. Part B Eng..

[35] Long Y., Zhang R., Huang J., Wang J., Zhang J., Rayand N., Hu G.-H., Yang J., Zhu J. (2017). Retroreflection in binary bio-based PLA/PBF blends. Polymer.

[36] Long Y., Zhang R., Huang J., Wang J., Jiang Y., Hu G.-H., Yang J., Zhu J. (2017). Tensile Property Balanced and Gas Barrier Improved Poly(lactic acid) by Blending with Biobased Poly(butylene 2,5-furan dicarboxylate). ACS Sustain. Chem. Eng..

[37] Poulopoulou N., Kasmi N., Bikiaris D.N., Papageorgiou D.G., Floudas G., Papageorgiou G.Z. (2018). Sustainable Polymers from Renewable Resources: Polymer Blends of Furan-Based Polyesters. Macromol. Mater. Eng..

[38] Poulopoulou N., Kasmi N., Siampani M., Terzopoulou Z., Bikiaris D.N., Achilias D.S., Papageorgiou D.G., Papageorgiou G.Z. (2019). Exploring Next-Generation Engineering Bioplastics: Poly(alkylene furanoate)/Poly(alkylene terephthalate) (PAF/PAT) Blends. Polymers.

[39] Papageorgiou G.Z., Tsanaktsis V., Bikiaris D.N. (2014). Synthesis of Poly(ethylene furandicarboxylate) polyester using monomers derived from renewable resources. Thermal behavior comparison with PET and PEN. Phys. Chem. Chem. Phys..

[40] Papageorgiou G.Z., Papageorgiou D.G., Tsanaktsis V., Bikiaris D.N. (2015). Synthesis of the bio-based polyester poly(propylene 2,5-furandicarboxylate). Comparison of thermal behavior and solid statestructure with its terephthalate and naphthalate homologues. Polymer.

[41] Safapour S., Seyed-Esfahani M., Auriemma F., Ruiz de Ballesteros O., Vollaro P., Di Girolamo R., De Rosa C., Khosroshahi A. (2010). Reactive blending as a tool for obtaining poly(ethylene terephthalate)-based engineering materials with tailored properties. Polymer.

[42] Sonchaeng U., Iñiguez-Franco F., Auras R., Selke S., Rubino M., Lim L.-T. (2018). Poly(lactic acid) mass transfer properties. Prog. Polym. Sci..

[43] Van Berkel J.-G., Guigo N., Visser H.A., Sbirrazzuoli N. (2018). Chain Structure and Molecular Weight Dependent Mechanics of Poly(ethylene 2,5-furandicarboxylate) Compared to Poly(ethylene terephthalate). Macromolecules.

[44] Achilias D.S., Papageorgiou G.Z., Karayannidis G.P. (2004). Isothermal and Nonisothermal Crystallization Kinetics of Poly(propylene terephthalate). J. Polym. Sci. Part B Polym. Phys..

[45] Papageorgiou G.Z., Achilias D.S., Karayannidis G.P., Bikiaris D.N., Roupakias C., Litsardakis G. (2006). Step-scan TMDSC and high rate DSC study of themultiple melting behavior of poly(1,3-propylene terephthalate). Eur. Polym. J..

[46] Papageorgiou G.Z., Karayannidis G.P. (2001). Crystallization and melting behaviour of poly(butylene naphthalene-2,6-dicarboxylate). Polymer.

[47] Papageorgiou D.G., Bikiaris D.N., Papageorgiou G.Z. (2018). Synthesis and controlled crystallization of in situ prepared poly(butylene-2,6-naphthalate) nanocomposites. CrystEngComm.

[48] Terzopoulou Z., Kasmi N., Tsanaktsis V., Doulakas N., Bikiaris D.N., Achilias D.S., Papageorgiou G.Z. (2017). Synthesis and Characterization of Bio-Based Polyesters: Poly(2-methyl-1,3-propylene-2,5-furanoate), Poly(isosorbide-2,5-furanoate), Poly(1,4-cyclohexanedimethylene-2,5-furanoate). Materials.

[49] Van Krevelen D.W. (2019). Properties of Polymers: Their Correlation with Chemical Structure: Their Numerical Estimation and Prediction from Additive Group Contributions.

